# Medium-term chest computed tomography (CT) follow-up of COVID-19 pneumonia patients after recovery to assess the rate of resolution and determine the potential predictors of persistent lung changes

**DOI:** 10.1186/s43055-021-00434-z

**Published:** 2021-02-16

**Authors:** Arshed Hussain Parry, Abdul Haseeb Wani, Naveed Nazir Shah, Majid Jehangir

**Affiliations:** 1 Department of Radiodiagnosis, Sher-i-Kashmir Institute of Medical Sciences, Srinagar, Jammu & Kashmir India; 2 Department of Radiodiagnosis, Government Medical College, Srinagar, Jammu & Kashmir India; 3 Department of Respiratory Medicine, Chest Disease Hospital, Srinagar, Jammu & Kashmir India

**Keywords:** COVID-19 survivors, Medium-term follow-up, Post-COVID-19 lung changes, Post-COVID-19 fibrosis, Persistent lung abnormalities, Pulmonary sequelae of COVID-19

## Abstract

**Background:**

The data on medium-term follow-up of coronavirus disease-19 (COVID-19) pneumonia survivors is scarce. Medium-term follow-up will generate knowledge and help in devising a structured follow-up plan and to facilitate enrolment in clinical trials assessing the role of antifibrotic drugs in modifying the course of disease in order to avert long-term pulmonary sequelae of disease. The study was aimed to evaluate the lung findings on a medium-term follow-up (3 months or more) chest computed tomography (CT) in COVID-19 pneumonia survivors, assess the rate of resolution or persistence of lung abnormalities and to identify the initial demographic, clinical, and imaging characteristics that could potentially predict the persistence of lung abnormalities on follow-up.

**Results:**

Out of the total study cohort of 81 patients, 46 (56.8%) demonstrated complete resolution of lung findings and the remaining 35 (43.2%) had residual lung opacities on follow-up CT. The most common type of residual abnormality was ground glass opacity (GGO) (16/35; 45.7%), followed by parenchymal bands (9/35; 25.7%), mixed pattern of GGO and parenchymal bands (6/35; 17.2%), bronchiectasis (6/35; 17.2%), and interlobular septal thickening (4/35; 11.4%). Patients with residual abnormalities were older, had higher BMI, more comorbidities, lower SpO2, longer hospital stay, higher rate of intensive care unit (ICU) admission, higher WBC count, a higher CT severity score, and lower rate of steroid administration with all *p* values < 0.05.

**Conclusion:**

Nearly half of post-COVID-19 survivors had residual lung abnormalities after ≥ 3 months of follow-up. Certain clinico-radiological characteristics have the potential to identify the individuals at risk of having residual lung abnormalities on medium-term follow-up.

## Background

Coronavirus disease 19 (COVID-19) has caused a major global health crisis. The disease which was declared as a pandemic by World Health Organization (WHO) on March 11, 2020 has infected an estimated 57.8 million till 21 November, 2020 and has claimed over 1.37 million lives across the world [1].

The diagnosis of COVID-19 hinges on the detection of viral nucleic acid in the respiratory secretions using reverse transcriptase polymerase chain reaction (RT-PCR). The consensus guidelines from various radiological societies across the world have discouraged the routine use of chest computed tomography (CT) for establishing the diagnosis of COVID-19. However, performance of CT is essential in a subset of patients with severe disease and those showing respiratory deterioration during the course of illness. CT is also needed to monitor the course of disease or response to therapy [2–4].

The survivors of COVID-19 show varied clinical course after the initial recovery. Some patients recover fully with no residual symptoms or functional impairment; however, some experience persistent symptoms or functional impairment after the initial recovery [5].

As the long-term consequences of COVID-19 are not fully known at present, the data from previous coronavirus infections may provide useful insights as there may be important parallels between COVID-19 and its close “cousins” severe acute respiratory syndrome (SARS) and Middle East respiratory syndrome (MERS) which caused the outbreaks at 2003 and 2012, respectively.

In a longitudinal study of SARS survivors, 36% of patients had residual lung abnormalities at 3 months on chest X-ray (CXR) which decreased marginally to 30% at 6 months. When the radiographic abnormalities were correlated with pulmonary function tests, it was found that 16% of patients had impaired diffusion capacity of lungs (TLco) at 6 months, thus implying that residual radiological abnormalities were physiologically relevant [6]. Similarly, in MERS survivors, 36% of patients had residual lung abnormalities in radiographs after a follow-up of 1 to 8 months [7].

The preliminary reports suggest that pulmonary parenchymal abnormalities do not completely resolve in all the survivors of COVID-19, and in some, the initial inflammation progresses into lung fibrosis [5]. A few published studies have assessed the temporal course of pulmonary findings in COVID-19 pneumonia, but they have mainly focused on the short-term changes in pulmonary opacities, usually up to 4 weeks [8–11].

Understanding the intermediate-term lung changes on CT may help in the identification of patients at risk of long-term COVID-19-induced lung compromise which would prompt initiation of appropriate treatment. It may also serve as a basis for selecting patients for future trials involving antifibrotic therapies.

The present study was aimed to determine the rate of complete radiological resolution on medium-term follow-up (3 months or more) and to identify the individuals who are at risk of developing long-term pulmonary sequelae.

## Methods

### Study design and patient cohort

This was a retrospective study conducted at a designated COVID-19 Care Centre in India. Institutional Ethical Committee (IEC) approval was obtained. The requirement for patient’s informed consent was waived. RT-PCR confirmed COVID-19 patients who had undergone chest CT during the initial or active phase of illness and at least one follow-up CT with a gap of at least 3 months between the two scans were enrolled in the study. If the patient had undergone more than one CT scan during the initial illness, then the CT with highest percentage of total lung involvement (highest CT severity score) was selected. The main indications for follow-up CT included persistent symptoms or functional impairment or persistent significant lung findings on follow-up chest X-ray (CXR). In some cases, follow-up scans were performed on the relentless insistence of patients who were concerned about the sequelae of COVID-19.

The relevant demographic characteristics, clinical history, laboratory findings, pharmacological treatment received, and chest CT findings were collected and analyzed retrospectively.

### Data collection

Demographic characteristics like age, gender, preexisting health conditions (hypertension, diabetes mellitus, chronic kidney disease (CKD), chronic liver disease (CLD), chronic obstructive pulmonary disease (COPD), malignancy, immunosuppression), and smoking history were documented. Clinical characteristics including main symptoms during initial illness and at the time of follow-up, percutaneous oxygen saturation (SpO2), and body measurements to calculate body mass index (BMI) were recorded. Laboratory indices including white blood cell count (WBC), neutrophil to lymphocyte ratio (NLR), C-reactive protein (CRP), and lactate dehydrogenase (LDH) were collected. Important management and therapeutic data including outpatient vs inpatient, admission to intensive care unit (ICU), intubation, and the pharmacological treatment received were recorded.

#### CT acquisition protocol and image interpretation

All CT scans were performed on 16-Slice Siemens SOMATOM, Emotion Multidetector CT. Scans were acquired in a single breath-hold after setting up the patient in a head-first supine position in the CT gantry. The following scanning parameters were used: slice thickness 1–1.5 mm, tube voltage 100–120 kVp, tube current of 90–130 mAs, and a beam pitch of 1.5. The tube current was regulated by an automatic exposure control system. Images were reconstructed using reconstruction increment of 0.7 mm into a slice thickness of 1 mm. The images were viewed in lung window settings (width of 1200–1600 HU and centering of − 500 to – 600 HU) and mediastinal window (width of 300–400 HU and centering of 40 HU).

Each patient had two sets of images (initial and follow-up). The CT scans were anonymized by replacing patient names with a code number by a CT technologist to make the readers blind to avoid possible bias. The CT images were independently analyzed by two experienced radiologists (P.A and W.A with 9 and 8 years of experience, respectively) blinded to the clinical data. In case of any disagreements between the two primary interpreting radiologists, a third radiologist (J.M, with more than 20 years of experience) adjudicated the final decision.

The following CT imaging characteristics were studied: (1) presence or absence of lung opacities; (2) distribution of lung opacities: unilateral vs bilateral lung involvement; (3) number of lobes affected; (5) dominant type of lung opacity: ground glass opacity (GGO), consolidation, mixed pattern of GGO, and consolidation and linear/curvilinear opacities.

Pulmonary opacities were categorized using the Fleischner Society glossary of terms for thoracic imaging [12]. To estimate the extent of lung involvement, a semi quantitative scoring system based on the visual calculation of percentage area of pulmonary opacification was used [13]. Each lung lobe was visually scored from 0 to 5 as follows: no involvement (score 0), less than 5% involvement (score 1), 5–25% involvement (score 2), 26–50% involvement (score 3), 51–75% involvement (score 4) and 76–100% involvement (score 5). The scores of all five lobes were summed up to obtain a total CT severity score ranging from 0 (no involvement) to 25 (maximum involvement).

The interpretation of follow-up CT scans allowed categorization of patients into two groups based on the radiological clearance or persistence of pulmonary opacities: (1) patients with complete resolution of pulmonary opacities and (2) patients with residual pulmonary opacities.

## Statistical analysis

Data were analyzed using the Statistical Package for the Social Sciences (SPSS Inc., Chicago, IL, version 21.0). Continuous variables were expressed as means and standard deviations. Categorical variables were expressed as counts and percentages. Fisher’s exact test was used to examine the categorical variables. Two sample student *t* test was used for comparison of continuous variables when the data was normally distributed, while Mann–Whitney U test was used when the data was not normally distributed. A *p* value less than 0.05 was considered statistically significant.

## Results

The study included 81 patients comprising of 50 (61.7%) males and 31 (38.3%) females with a mean age of 51.8 ± 11.7 years (range 32–69 years). Based on the disappearance or persistence of pulmonary opacities on follow-up CT, patients were divided into two groups. A comparison of demographic, clinical, laboratory, therapeutic, and CT characteristics between the two groups is listed in Tables 1 and 2.

**Table 1:** Comparison of demographic, clinical, laboratory and therapeutic data between the two follow-up groups

Parameter	Complete radiological resolution on follow-up CT (*n* = 46; 56.8%)	Residual lung findings on follow-up CT (*n* = 35; 43.2%)	*p* value
Age (Mean ± SD)	45.8 ± 13.8	59.6 ± 9.3	0.01858
Sex	M = 29 (63%) F = 17 (37%)	M = 21 (60%) F = 14 (40%)	0.8205
BMI			
18–25	34 (74%)	14 (40%)	0.003
> 25	12 (26%)	21 (60%)	
Smoking history			
Smoker	13 (28.3%)	18 (51.4%)	0.6497
Non-smoker	33 (71.7%)	17 (48.6%)	
Comorbidity			
Absent	26 (56.5%)	7 (20%)	
Present	20 (43.5%)	28 (80%)	
Diabetes mellitus	6	8	
Hypertension	7	7	
COPD	4	5	0.0013
CKD	1	2	
CLD	1	2	
CAD	1	1	
Malignancy	0	2	
Immune suppression	0	1	
Hospitalization			
Inpatient	34 (74%)	31 (88.6%)	0.1583
Outpatient	12 (26%)	4 (11.4%)	
In-ward hospitalization	43 (93.5%)	25 (71.4%)	0.0126
ICU admission	3 (6.5%)	10 (28.6%)	
Duration of hospitalization			
(Mean days ± SD)	7.6 ± 2.3	11.2 ± 4.1	0.000332
Initial percutaneous oxygen saturation (%)			
(Mean ± SD)	92.3 ± 3.8	88.1 ± 2.2	0.00132
Initial WBC count			
×10^3^ (Mean ± SD)	5343 ± 1456.4	7987 ± 2465.8	0.00105
NLR	2.5 ± 1.1	2.8 ± 1.3	0.609
LDH, IU/l			
(Mean ± SD)	697.7 ± 198.8	743.8 ± 211.4	0.6921
CRP, mg/l			
(Mean ± SD)	34.6 ± 17.6	41.1 ± 19.8	0.4554
Pharmacological treatment			
Antiviral drugs	16 (34.8%)	13 (37.1%)	1
Hydroxychloroquine	12 (26%)	17 (48.6%)	0.63
Steroids	39 (84.8%)	18 (51.4%)	0.0007
Persistent symptoms on follow-up	30 (65.2%)	24 (68.5%)	0.8148

*CT* computed tomography, *BMI* body mass index, *NLR* neutrophil to lymphocyte ratio, *CRP* C-reactive protein, *LDH* lactate dehydrogenase, *WBC* white blood cell count, *COPD* chronic obstructive pulmonary disease, *CKD* chronic kidney disease, *CLD* chronic liver disease, *CAD* coronary artery disease

**Table 2:** Comparison of initial and follow-up CT between the two follow-up groups

Parameter	Complete radiological resolution on follow-up CT (*n* = 46; 56.8%)	Residual lung findings on follow-up CT (*n* = 35; 43.2%)	*p* value
Laterality of lung involvement on initial CT			
Bilateral	33 (71.8%)	29 (82.8%)	0.2962
Unilateral	13(28.2%)	6 (17.2%)	
Number of lobes involved on initial CT	3 ± 1.3	3.4 ± 1.4	0.6351
Percentage of total lung involvement on initial CT (CT severity score)	8.1 ± 2.3	12.4 ± 3.2	0.03843
Dominant pulmonary opacity on initial CT			
Pure GGO	34 (73.9%)	24 (68.6%)	0.6266
Consolidation	4 (8.7%)	6 (17.1%)	0.3155
Mixed pattern	8 (17.4)	5 (14.3%)	0.7686
Dominant pulmonary opacity on follow-up CT			
GGO	N/A	16 (45.7%)	N/A
Linear/curvilinear parenchymal bands		9 (25.7%)	
Mixed pattern of GGO and parenchymal bands		6 (17.2%)	
Interlobular septal thickening		4 (11.4%)	
Bronchiectasis		6 (17.2%)	
Time lapse between initial and follow-up CT (Mean days ± SD)	101.4 ± 9.8	99.6 ± 8.7	0.4737

*CT* computed tomography, *GGO* ground glass opacity

**Fig. 1:**
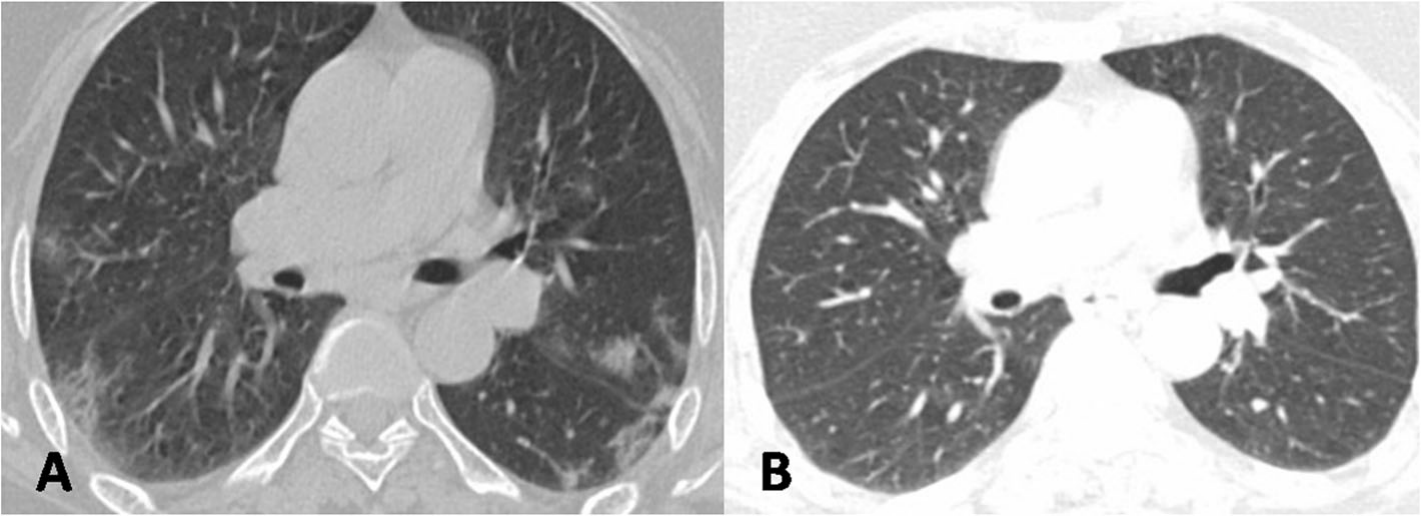
Axial chest CT image in lung window settings of a 49-year-old COVID-19 patient showing multifocal subpleural ground glass opacities (GGOs) in both lungs at the time of hospital admission (**a**). Follow-up CT performed 93 days after the first CT reveals complete resolution of GGOs (**b**)

The average time interval between the initial and follow-up CT was 100.6 days (range 90–111 days). The follow-up CT revealed complete resolution of pulmonary opacities in 46/81 (56.8%), whereas 35/81 (43.2%) demonstrated residual pulmonary opacities (Fig. 1). The mean time lag between the initial and follow-up CT scans was not statistically different between the two follow-up groups of complete radiological resolution and persistent lung opacities (*p* value = 0.473).

Patients who demonstrated residual pulmonary opacities on follow-up CT were older (59.6 ± 9.3 years) compared with the group demonstrating complete radiological resolution on follow-up CT (45.8 ± 13.8) (*p* value = 0.01858).

Patients who showed persistent lung opacities on follow-up CT were more frequently overweight or obese (21/35; 60%) compared with the group showing complete radiological resolution (12/46; 26%) with a statistically significant difference (*p* value = 0.003).

Though the group showing residual lung opacities had a higher number of smokers compared with the group showing complete radiological resolution (51 vs 28.3%), the difference did not however reach statistical significance (*p* value = 0.65).

With regards to the presence or absence of underlying comorbid illness, a statistically higher percentage of patients demonstrating residual lung opacities had an underlying comorbidity compared with the resolved group (80% vs 43.5%; *p* value = 0.0013).

No statistically significant difference was seen in the hospitalization rate between the resolved and residual groups (74 vs 88.6%) (*p* value = 0.1583). However, there was a significant difference in the rate of ICU admission between the groups of resolved and residual opacities (6.5 vs 28.6%) (*p* value = 0.0126).

The duration of hospital stay was significantly higher in the group demonstrating residual pulmonary opacities compared with the group demonstrating complete radiological resolution (11.2 ± 4.1 days vs 7.6 ± 2.3 days) (*p* value = 0.00033).

Among the laboratory parameters initial WBC count was significantly different between the two groups of residual and resolved opacities (7987 × 10^3^ ± 2465.8 vs 5343 × 10^3^ ± 1456.4) (*p* value = 0.00105). However, NLR ratio was not statistically different between the two groups. Initial LDH and CRP levels did not differ significantly between the two follow-up groups of resolved and residual opacities (*p* values of 0.455 and 0.69, respectively).

The percutaneous oxygen saturation was significantly more in patients who subsequently demonstrated complete radiological resolution compared with the group demonstrating residual lung opacities (92.3 ± 3.2% vs 88.1 ± 2.2%) (*p* value = 0.0013).

With regards to the pharmacological treatment, those receiving steroids (dexamethasone or methylprednisolone) showed complete radiological resolution more frequently (39/46; 84.8% vs 18/35; 51.4%) (*p* value = 0.0007) compared with the group showing persistence of lung opacities. However, hydroxychloroquine and antiviral (remedesevir) administered groups did not show any significant difference with regards to disappearance or persistence of lung opacities (*p* value = 1 and 0.63, respectively).

Patients who demonstrated residual lung opacities on follow-up CT had a significantly higher percentage of total lung involvement (CT severity score 12.4 ± 3.2) on initial CT compared with the group demonstrating complete radiological resolution (CT severity score 8.1 ± 2.3) (*p* value = 0.03843).

The type of dominant pulmonary opacities on initial CT did not determine the future resolution or persistence of opacities as GGO was the commonest lung opacity observed in 73.9% of the resolved group and 68.6% of the residual group (*p* value = 0.6266).

Similarly, unilateral or bilateral lung involvement or the number of lung lobes involved on initial CT did not differ significantly between the two groups of residual or resolved opacities (*p* values = 0.2962 and 0.6351, respectively).

Only 33.3% (27/81) patients were completely free of any symptoms on follow-up, while 63.7% (54/81) reported one or more persistent symptoms. The commonest symptoms reported were fatigue (24/54; 44.4%), chest discomfort or pain (16/54; 29.6%), dyspnea (14/54; 25.9%) and joint pains (6/54; 11.1%). However, a comparison of persistent symptoms between two follow-up groups of resolved and residual lung findings did not show any statistically significant difference between them (Table 1).

**Fig. 2:**
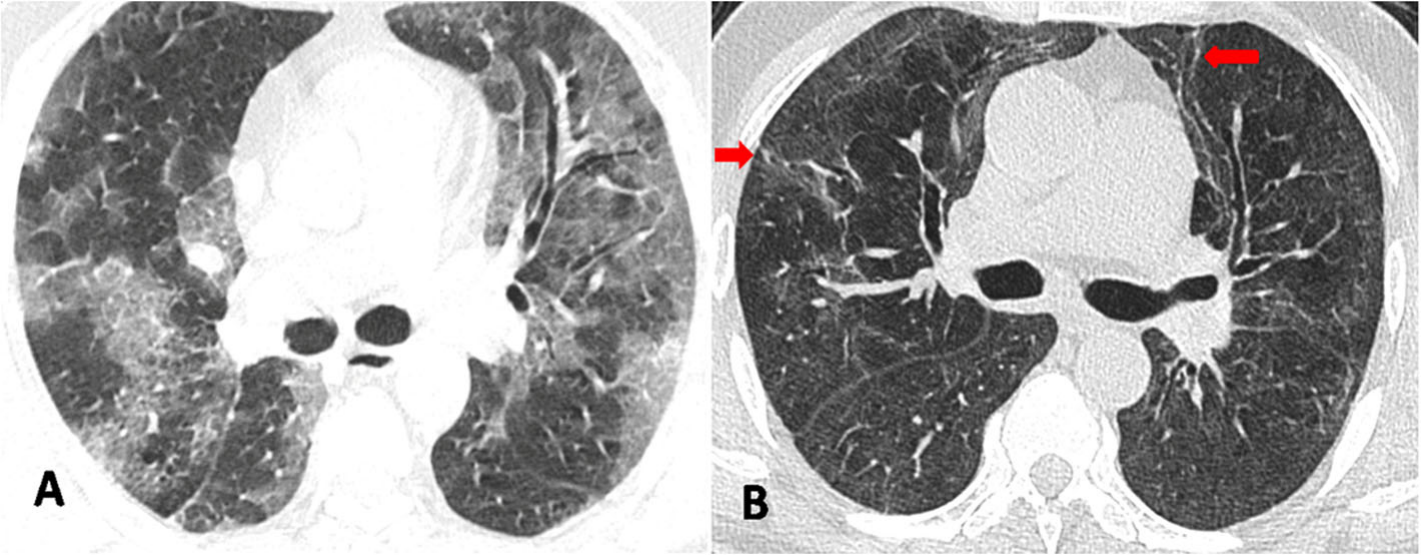
Axial chest CT image in lung window settings of a 52-year-old COVID-19 patient showing diffuse ground glass opacification in both lungs just below the level of the carina at the time of hospital admission (**a**). Follow-up CT performed 97 days after the first CT reveals residual faint ground glass opacities in both lungs with architectural distortion and bronchiectasis (red arrows) (**b**)

**Fig. 3:**
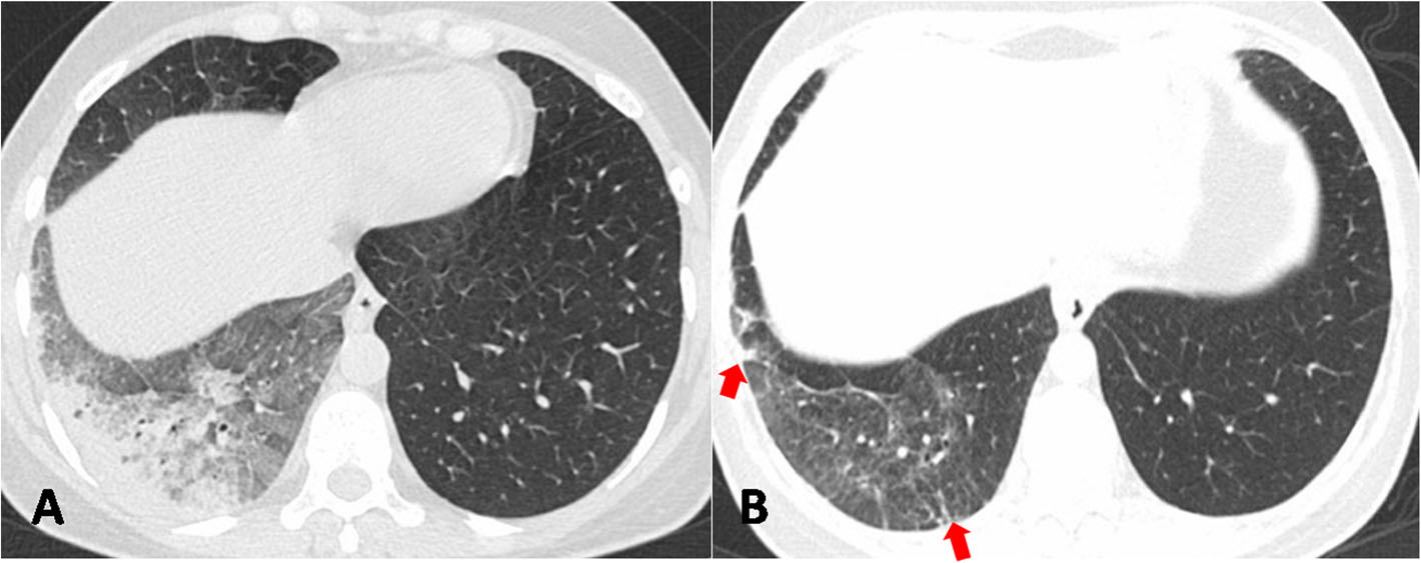
Axial chest CT image in lung window settings of a 36-year-old COVID-19 patient showing a mixed GGO and consolidation in right lower lobe at admission (**a**). Follow-up CT performed 92 days later reveals residual GGO with subpleural parenchymal bands (red arrows) (**b**)

**Fig. 4:**
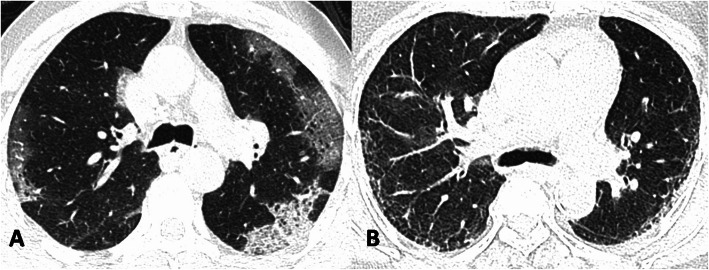
Axial chest CT image in lung window settings of a 51-year-old COVID-19 patient at the level of carina obtained at admission reveals elongated GGOs in the subpleural lung (**a**). Follow-up CT performed 107 days later reveals residual faint GGOs with interlobular septal thickening in the subpleural lung (**b**)

Dominant pulmonary opacities observed on follow-up CT included GGO (16/35; 45.7%), linear/curvilinear subpleural parenchymal bands (9/35 25.7%), mixed pattern of GGO and parenchymal bands (6/35; 17.2%), and interlobular septal thickening (4/35; 11.4%) (Figs. 2, 3, and 4). Bronchiectasis was observed in (6/35; 17.2%) (Fig. 2).

## Discussion

As COVID-19 continues its relentless march across the world, follow-up data on survivors of infection has begun to emerge. However, the majority of data available at present focuses on the short-term sequelae of infection and data on medium-term radiological findings are scarce. Persistent clinical symptoms were reported in 87% post-COVID-19 survivors after a mean follow-up of 2 months in an Italian COVID-19 survivor cohort [14]. Ye et al. [15] reported complete radiological resolution in 37% of survivors, whereas persistent abnormalities consisting predominantly of subpleural parenchymal bands were observed in 47% survivors after a mean follow-up of 64 days after symptom onset.

You et al. [16] recorded residual lung abnormalities commonly in post-COVID-19 survivors including GGO in 73% and pulmonary fibrosis in 26% after a mean of 40 days after discharge from hospital.

Similarly, in a clinico-radiologic follow-up of a cohort of 55 Chinese patients after recovery, it was found that 63% had persistent symptoms and 71% had residual lung opacities including interstitial thickening in 27% after a mean interval of 3 months [5]. The preliminary prepublication findings from a prospective observational cohort presented in European Respiratory Society (ESR) reported lung abnormalities on CT in 88% at 6 weeks which dropped to 56% after 12 weeks [17].

The results of the present study suggest that a substantial proportion of patients show residual lung opacities at medium-term follow-up on CT. The persistent lung opacities are potentially suggestive of permanent lung damage which eventually heals with fibrosis. Viral pneumonias may recover without any residual lung damage or fibrosis. However, there are many viral infections of lungs that have been documented to leave the trail of destruction behind in the form of lung fibrosis. H1N1 pneumonia is occasionally complicated by pulmonary fibrosis [17]. However, the percentage of patients showing residual lung abnormalities post recovery have been reported in 22% survivors of H7N9, 33% of MERS, and 38% of SARS after a mean time interval of 6, 2–8, and 6 months, respectively [18, 19]. In comparison, 45% of post-COVID-19 survivors showed residual lung abnormalities after a mean follow-up time of more than 3months.

There were many important demographic, clinical, laboratory, and imaging characteristics that were more frequently associated with persistence of residual lung changes. Older age, obesity, preexisting comorbidity, lower oxygen saturation, prolonged hospital stay, ICU admission, and a higher WBC count during acute phase of illness were more commonly associated with residual lung changes on follow-up. Similarly, patients having a more severe lung involvement during initial phase of the disease, as assessed by semi quantitative CT severity score were more likely to have residual lung findings on medium-term follow-up. The more severe the lung injury the greater is the inflammatory response which in turn incites fibroblastic response leading to pulmonary fibrosis [20]. Administration of steroids was associated with lesser probability of having residual lung abnormalities on follow-up which suggests that steroid may have a role in modifying the course of illness and preventing permanent lung damage. A biphasic model of the disease has been proposed to understand the course of COVID-19 [21]. While the symptoms and respiratory dysfunction in the first phase are related to viral replication, in the second phase the symptoms are predominantly due to inflammatory response [21]. Anti-inflammatory agents like steroids have been found to improve the outcome in COVID-19 pneumonia by checking the inflammatory response and the same mechanism may hasten the clearance of lung opacities [21–23]. At this stage, however, it is admittedly too early to extrapolate the potential role of steroids in dissipation or resolution of lung opacities in COVID-19 based on the limited data presented in our study. Further prospective studies with large number of patients should be performed to validate the findings of our study.

Though the optimal time for follow-up imaging to assess for radiological clearance is not known, current guidelines of British Thoracic Society recommend a 12-week follow-up to provide adequate time for lung abnormalities to resolve which also ensures that non-resolving findings are addressed sufficiently early [19]. A follow-up CT is not recommended for patients without pneumonia during the initial active phase of disease nor in patients who show complete resolution of lung findings on follow-up CXR at the time of discharge. High-resolution CT is indicated for evaluation of patients with persistent significant lung abnormalities on follow-up CXR and in patients with ongoing respiratory symptoms or physiological impairment [19, 24].

The medium-term follow-up data is envisaged to provide important information which will guide in devising a structured follow-up plan of COVID-19 survivors to mitigate the long-term sequelae of the disease. Whether the CT findings observed on medium-term follow-up resolve or lead to permanent pulmonary fibrosis will only become apparent with longer-term follow-up.

This study has some limitations. Firstly, only 81 patients were enrolled in the study. A large sample size is desirable to study the pulmonary sequelae of COVID-19. Secondly, we did not perform pulmonary function tests on follow-up which could give more precise information on the physiological impairment of the lungs. Third, we did not perform CT angiography or perfusion imaging to study the burden of residual pulmonary thrombosis. It is being increasingly suggested that pulmonary vascular sequelae may be an important long-term effect of COVID-19 and the residual clot burden can be demonstrated by CT perfusion imaging.

## Conclusion

Nearly half of the COVID-19 survivors had residual lung abnormalities after a time interval of 3 months or more. Older age, obesity, presence of preexisting comorbidity, longer duration of hospital stay, ICU admission, higher initial WBC count, a higher initial CT severity score, and a lower rate of steroid administration are associated with more prevalence of residual lung abnormalities in COVID-19 survivors.

## Data Availability

The data is available with the first three authors.

## Abbreviations

COVID-19: Coronavirus disease-19
RT-PCT: Reverse transcriptase polymerase chain reaction
CT: Computed tomography
CXR: Chest X-ray
SARS: Severe acute respiratory syndrome
MERS: Middle East respiratory syndrome
